# Visual search as a cognitive marker to discriminate progressive supranuclear palsy from Parkinson’s disease

**DOI:** 10.1093/braincomms/fcag422

**Published:** 2026-11-03

**Authors:** Alexis Cheviet, Alison R Lane, Anthony P Atkinson, Uma Nath, Claire McDonald, Louise Wiblin, Richard W Walker, Daniel T Smith

**Affiliations:** Department of Psychology, University of Durham, Durham DH1 3LE, UK; Department of Psychology, University of Durham, Durham DH1 3LE, UK; Department of Psychology, University of Durham, Durham DH1 3LE, UK; Department of Neurology, Sunderland Royal Hospital, South Tyneside and Sunderland NHS Foundation Trust, Sunderland SR4 7TP, UK; AGE Research Group, Translational and Clinical Research Institute, Faculty of Medical Sciences, Newcastle University, Newcastle upon Tyne NE4 5PL, UK; NIHR Newcastle Biomedical Research Centre, Newcastle upon Tyne Hospitals NHS Foundation Trust, Westgate Rd, Newcastle upon Tyne NE4 5PL, UK; Cumbria, Northumberland, Tyne and Wear NHS Foundation Trust, Jubilee Road, Gosforth, Newcastle upon Tyne NE3 3XT, UK; Queen Elizabeth Hospital, Gateshead Health NHS Trust, Gateshead NE9 6SX, UK; Department of Neurology, South Tees Hospitals NHS Foundation Trust, Middlesbrough TS4 3BW, UK; Northumbria Healthcare NHS Foundation Trust, North Tyneside General Hospital, North Shields NE29 8NH, UK; Population Health Sciences Institute, Newcastle University, Newcastle upon Tyne NE4 5PL, UK; Department of Psychology, University of Durham, Durham DH1 3LE, UK

**Keywords:** vision, cognition, attention, progressive supranuclear palsy, Parkinson’s disease

## Abstract

Early and accurate diagnosis of progressive supranuclear palsy is a high priority for both patients and clinicians. Nearly half of individuals with progressive supranuclear palsy are initially given an alternative diagnosis—most commonly Parkinson’s disease—and the correct diagnosis of progressive supranuclear palsy is delayed by an average of 3 years compared with Parkinson’s disease. Problems with cognition arise early in the progression of progressive supranuclear palsy, including profoundly disturbed visual search. This search deficit is likely caused by a combination of oculomotor, visuospatial, attentional and executive dysfunction, which is not observed in Parkinson’s disease. Since assessment of visual search is not routinely included in clinical testing for progressive supranuclear palsy, it may represent a promising method for differentiating these movement disorders. However, few studies have systematically compared visual search in progressive supranuclear palsy and Parkinson’s disease, so the extent to which search deficits can reliably differentiate the two diseases is unknown. To address this issue, patients with progressive supranuclear palsy (*N* = 28), Parkinson’s disease (*N* = 28) and age-matched controls (*N* = 27) completed four tests of visuospatial attention: an easy feature search task, a difficult feature search task, a Conjunction search task and a paper-and-pencil cancellation task (the Bells test). We measured perceptual sensitivity (*d′*), search time and detection accuracy. Search efficiency in the Bells test was also modelled using the Quality of Search score. In addition, patients completed tests of oculomotor system integrity, the Montreal Cognitive Assessment and the National Eye Institute Visual Function Questionnaire 25. As predicted, progressive supranuclear palsy was associated with a profound search deficit: search was slow and highly disorganized on all tests. Furthermore, reflexive orienting along the vertical axis was impaired on the easy feature search. Critically, receiver operating characteristic analyses revealed that the Quality of Search score derived from the Bells test classified progressive supranuclear palsy with a sensitivity of 82%, a specificity of 96% and an area under the curve of 0.90. Given that the Bells test is fast to administer (∼5 min), free, relatively simple to score and already widely used in neuropsychology assessment, it has the potential to be a highly effective addition to the battery of tests used in the differential diagnosis of progressive supranuclear palsy.

## Teaser

Delayed diagnosis is common in progressive supranuclear palsy. Here, Smith *et al*. report that the Bells test (a test of visuospatial attention) discriminated progressive supranuclear palsy and Parkinson’s disease with excellent sensitivity and specificity. Highly inefficient visual search may be a valuable diagnostic marker for progressive supranuclear palsy.

## Graphical Abstract

**Graphical Abstract:**
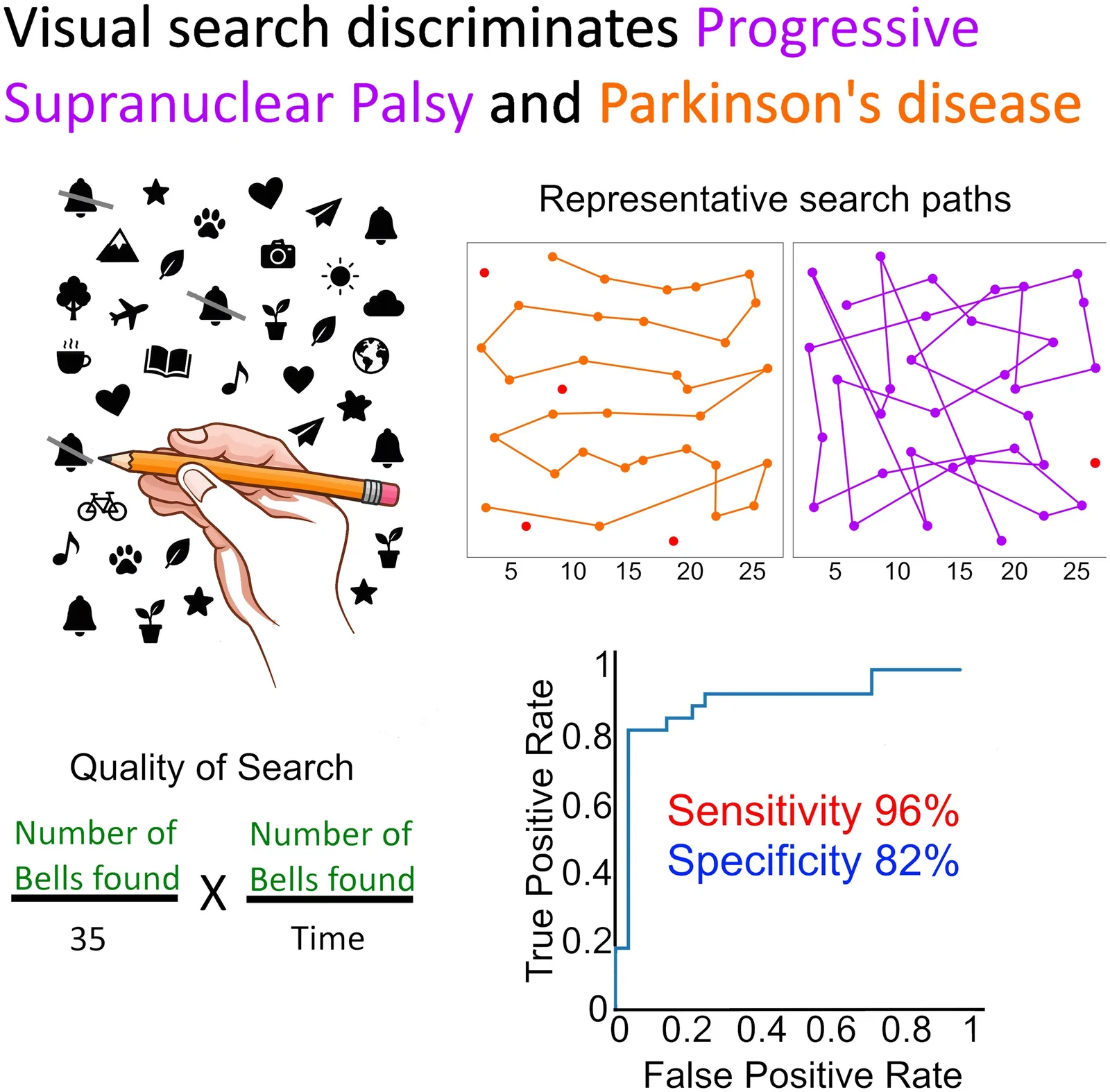

## Introduction

Progressive supranuclear palsy (PSP) is a rare and devastating neurodegenerative disease.^1-3^ It belongs to the 4R-tauopathies, which are characterized by substantial neuronal loss and gliosis due to the accumulation of four-repeat isoform tau protein aggregates.^4^ Depositions mainly affect the brainstem, the basal ganglia and, to a lesser extent, the cortical areas.^5,6^ Diagnosis of PSP is challenging because specific biomarkers have yet to be identified,^7^ and diagnostic criteria have evolved significantly since the original description in 1964.^8^ The current Movement Disorder Society criteria for Progressive Supranuclear Palsy (MDS-PSP)^9^ identify four core clinical domains: ocular motor dysfunction, postural instability, akinesia and cognitive deficits (including speech/language disorder, frontal/behavioural presentation and corticobasal syndrome). The MDS-PSP stratifies diagnosis into three degrees of certainty (probable PSP, possible PSP and suggestive of PSP) based on specific combinations of clinical features within these domains. The sensitivity of MDS-PSP within the first 3 years ranges from 64^10^ to 84%,^11^ but the corresponding specificity of 34.2–75.9%^10,11^ indicates that additional markers are required to further improve diagnosis. Delayed diagnosis of PSP is common, with at least 50% of patients initially receiving an alternative diagnosis, most frequently Parkinson’s disease.^12^ As a result, development of new approaches to diagnosis is a high priority for patients, carers and clinicians.

One promising avenue for exploration is the use of cognitive markers for PSP. Clinically, problems with speech, memory, attention and executive function are visible from 3 to 10 years before formal diagnosis,^13-16^ with cognitive symptoms being significantly worse than in patients with Parkinson’s disease.^14^ At a systems level, atrophy of the midbrain and/or frontal lobe has been identified as potentially the earliest structural alterations across most PSP subtypes,^17-20^ with these regions playing a dual role in both motor and cognitive processes.^21-25^ Indeed, midbrain structures such as the superior colliculus have important roles in both oculomotor function and visuospatial cognition.^26-29^ Consistent with this functional overlap, the characteristic vertical–horizontal anisotropy in eye movements seen in PSP is also observed in reflexive orienting of visuospatial attention^30-33^ and visuospatial short-term memory.^33,34^ Atrophy of the frontal cortex has been associated with apathy,^35,36^ social cognition deficits,^37^ speech/language disorder^17,38,39^ and dysexecutive syndrome,^17,36,40,41^ which have been investigated using a variety of cognitive assessments.^40-45^ For example, the Frontal Assessment Battery^17,46-49^ has shown potential to discriminate PSP from Parkinson’s disease and multiple system atrophy—parkinsonian type [area under the curve (AUC) = 0.927].^47^ The Montreal Cognitive Assessment (MoCA)^50^ and Mini-Mental State Examination (MMSE)^51^ are also widely used. While the MoCA outperforms the MMSE,^52-54^ it does not effectively capture cognitive decline in PSP over time,^55-57^ and lower MoCA scores in PSP are largely driven by deficits in a limited subset of cognitive domains.^52^ There remains no clear consensus on the optimal neuropsychological tests to improve diagnosis.

Visual search test, in which a participant is instructed to find a target object among a set of distractor objects, is a cognitive test that engages saccadic eye movements,^58^ upper body limb movements,^59^ visuospatial attention^60,61^ and executive control.^62,63^ As it relies on three of the four core functional domains affected by PSP identified by the MDS-PSP, it appears a promising candidate for a cognitive test that would be sensitive to PSP. Consistent with this idea, patients with PSP have significantly slower search times than patients with Parkinson’s disease,^64^ especially when the target appears along the vertical axis compared with the horizontal axis,^31^ and show a striking deficit of ‘strategy adherence’ in multiple-target search tests.^56^ Parkinson’s disease is also associated with longer completion times^65^ and/or elevated error rates^66^ relative to non-diseased controls, but these deficits occur mostly in advanced-stage patients^67^ and are primarily due to sensorimotor, not cognitive factors.^68,69^

These data suggest that cognitive tests, and particularly visual search tasks, could be a valuable tool to discriminate PSP from Parkinson’s disease. However, while prior studies comparing visual search in these diseases clearly show large group-level differences on reaction time (RT) and search strategy, they do not evaluate the classification accuracy of these tests. Furthermore, there are several well-established methods for evaluating search efficiency in both single-target and multiple-target search tasks that provide a more sophisticated quantification of performance than either simple RT or binary classification into efficient/inefficient search style. More specifically, in single-target search tasks, analysis of search Slopes (the increase in RT associated with additional distractors) gives an estimate of search efficiency that controls for differences in motor movement time between groups. In multiple-target search, the Quality of Search (QoS) parameter estimates search efficiency,^70^ and inter-cancellation (IC) distance, intersection rate and best *R* have been proposed as measures of search organization or strategy.^62^ The present study tests the hypothesis that PSP is associated with a deficit of visual search and examines whether measures of search efficiency can discriminate between PwPSP and PwP with sufficient accuracy to have the potential to facilitate differential diagnosis of these diseases.

## Materials and methods

### Participants

Participants were 28 people with Parkinson’s disease (PwP; 12 females), 28 people with PSP (PwPSP; 11 females), and 27 age-matched controls (AMC; 13 females). Patients were recruited via outpatient clinics at four hospitals and two charities (PSPA and Parkinson’s UK). Table 1 presents demographic data for each group. Neurologists and geriatricians specializing in the diagnosis of movement disorders diagnosed participants according to routine clinical practice, using the UK Brain Bank criteria and the MDS-PSP diagnostic criteria where applicable. Where MDS-PSP criteria were used, a diagnosis of possible or probable PSP was the inclusion criterion. Exclusion criteria encompassed (i) inability to provide written informed consent; (ii) inability to perform the experiment (poor sitting ability, severe cognitive impairment, severe visual loss; note that the presence of gaze paralysis was not an exclusion criterion); (iii) presence of an active psychiatric illness; and (iv) history of alcohol abuse, head injury, stroke, epilepsy or other major physical illness. All participants gave written informed consent. The protocol was approved by the North East–Newcastle and North Tyneside 1 Research Ethics Committee (19/NE/0369) and conducted in accordance with the BPS Code of Ethics. Sample size was determined by an *a priori* power analysis based on a prior study.^33^ With α = 0.05 and power = 0.99, G*Power indicated that 27 participants per group were required.

**Table 1:** Demographics and neuropsychological characteristics of the participants

	Group			Test and value	*P*
	AMC (*N* = 27)	PwD (*N* = 28)	PwPSP (*N* = 28)		
Sex ratio (M:F)	14:13	16:12	17:11		
Medication^a^					
N04B: dopa agents	N/A	A(26) C(4) D(3) X(2)	A(25) B(2) D(2)		
N06DA02: donepezil	N/A	0	3		
Unmedicated	N/A	1	1		
Onset of symptoms	N/A	7.41 ± 3.84	3.85 ± 2.63	*t* = 3.74	<0.05
Diagnosis	N/A	5.83 ± 3.84	2.14 ± 2.02	*t* = 4.50	<0.05
Age	69.44 ± 7.77	67.36 ± 10.60	72.43 ± 7.45	*F*_(2,80)_ = 2.38	0.099
Education	13.78 ± 3.44	13.29 ± 3.40	12.68 ± 3.75	*F*_(2,80)_ = 0.67	0.52
Fatigue (pre)	8.80 ± 1.04	6.83 ± 1.85	6.47 ± 1.81	*F*_(2,80)_ = 18.18	<0.01^b^
Fatigue (post)	7.35 ± 1.48	5.85 ± 2.01	4.69 ± 2.04	*F*_(2,80)_ = 3.86	<0.05^c^
PDQ 39	N/A	28.59 ± 18.20	35.35 ± 17.82	*t* = 1.40	0.17
NEI-VFQ 25	94.87 ± 4.69	87.24 ± 10.71	67.36 ± 24.60	*F*_(2,80)_ = 24.35	<0.01^d^

N/A, not applicable. ^a^Medications are classified based on the Anatomical Therapeutic Chemical Classification system. N04B: dopaminergic agents and related subclasses (A: dopa and dopa derivatives; B: adamantane derivative; C: dopamine agonists; D: monoamine oxidase B inhibitors; X: other dopaminergic agents); N06DA refers to anticholinesterases, but only donepezil (N06DA02) was reported. ^b^Bonferroni-corrected *post hoc t*-tests analysing the main effects of pre-test Fatigue showed significant differences between PSP and AMC (*t* = 6.02, *P* < 0.01) and between Parkinson’s disease and AMC (*t* = 5.04, *P* < 0.01), but not between PwPSP and PwP(*t* = 0.74, *P* = 1). ^c^Bonferroni-corrected *post hoc* tests analysing the main effects of post-test Fatigue showed significant differences between PwPSP and AMC (*t* = 5.52, *P* < 0.01) and between PwP and AMC (*t* = 3.14, *P* < 0.01), but not between PwPSP and PwP (*t* = 2.12., *P* = 0.115). ^d^Bonferroni-corrected *post hoc* tests analysing the main effects of NEI-VFQ 25 showed significant differences between PwPSP and AMC (*t* = 5.81, *P* < 0.01), PwP and AMC (*t* = 3.44, *P* < 0.01), and PwPSP and PwP (*t* = 3.92, *P* = 0.01).

### General design

Computerized tasks were performed on a computer (screen dimensions: 39.5 × 19.5 cm) positioned ∼57 cm from the participant’s eyes. Experimental stimuli were generated using PsychoPy software and OpenSesame. Eye movements were recorded using a Tobii X3-120 system (sampling rate: 120 Hz; saccadic velocity threshold: 35°/s; acceleration threshold: 9500°/s^2^) after a three-point calibration phase along the horizontal meridian. Participants were instructed not to make any head movements during the recording phase.

#### Oculomotor tasks

##### Saccadic eye movements

Each trial began with a white fixation point displayed at the centre of a black screen. After a variable delay between 1200 and 1800 ms, the dot jumped into the periphery, and participants were instructed to move their line of sight to it. Once completed, they pressed a button to start the next trial after a 1500-ms inter-trial interval. Four runs of 11 saccades that increased in amplitude in 0.75° steps, starting with a 2° jump, were implemented, one for each direction (upward, downward, leftward and rightward). The dependent variable was the amplitude gain (ratio between saccadic amplitude and target eccentricity).

##### Smooth pursuit eye movements

Smooth pursuit eye movements (SPEMs) were elicited by a sinusoidal movement of a white dot (amplitude range ±7.88°). Four runs were implemented to test each meridian (vertical/horizontal) with two frequencies (0.3 or 0.6 Hz), resulting in peak velocities of 15 and 30°/s, respectively. Each run consisted of 11 cycles. The dependent variable was SPEM velocity gain.

#### Visual search tasks

##### Single-target search

Participants searched for a single target among a set of distracters while fixating the centre of the screen. There were three versions of the task: (i) Easy-Feature, where the target was a unique colour among homogeneous distractors; (ii) Hard-Feature, where the target was a mirror-reverse of the distractor items; and (iii) Conjunction, where the target was a unique combination of features among distractors that share one feature with the target. In the Easy- and Hard-Feature tasks **(**Fig. 1A and B), the distractors were blue ‘c’ shapes oriented at 315°, while the visual search targets were a yellow ‘c’ shape and a mirror-reversed (135°) blue ‘c’ shape, respectively. In the Conjunction task (Fig. 1C), the distractors were a blue forward-oriented ‘c’ shape and yellow backward-oriented ‘c’ shape, while the target was a yellow forward-oriented ‘c’ shape. There were eight conditions per task including two levels of Set Size (6 or 12 items), two levels of Axis (horizontal or vertical), and two levels of Target Presence (present or absent). Each condition consisted of 18 trials, leading to a total of 144 trials per task. Eight training trials were performed before each task.

**Figure 1:**
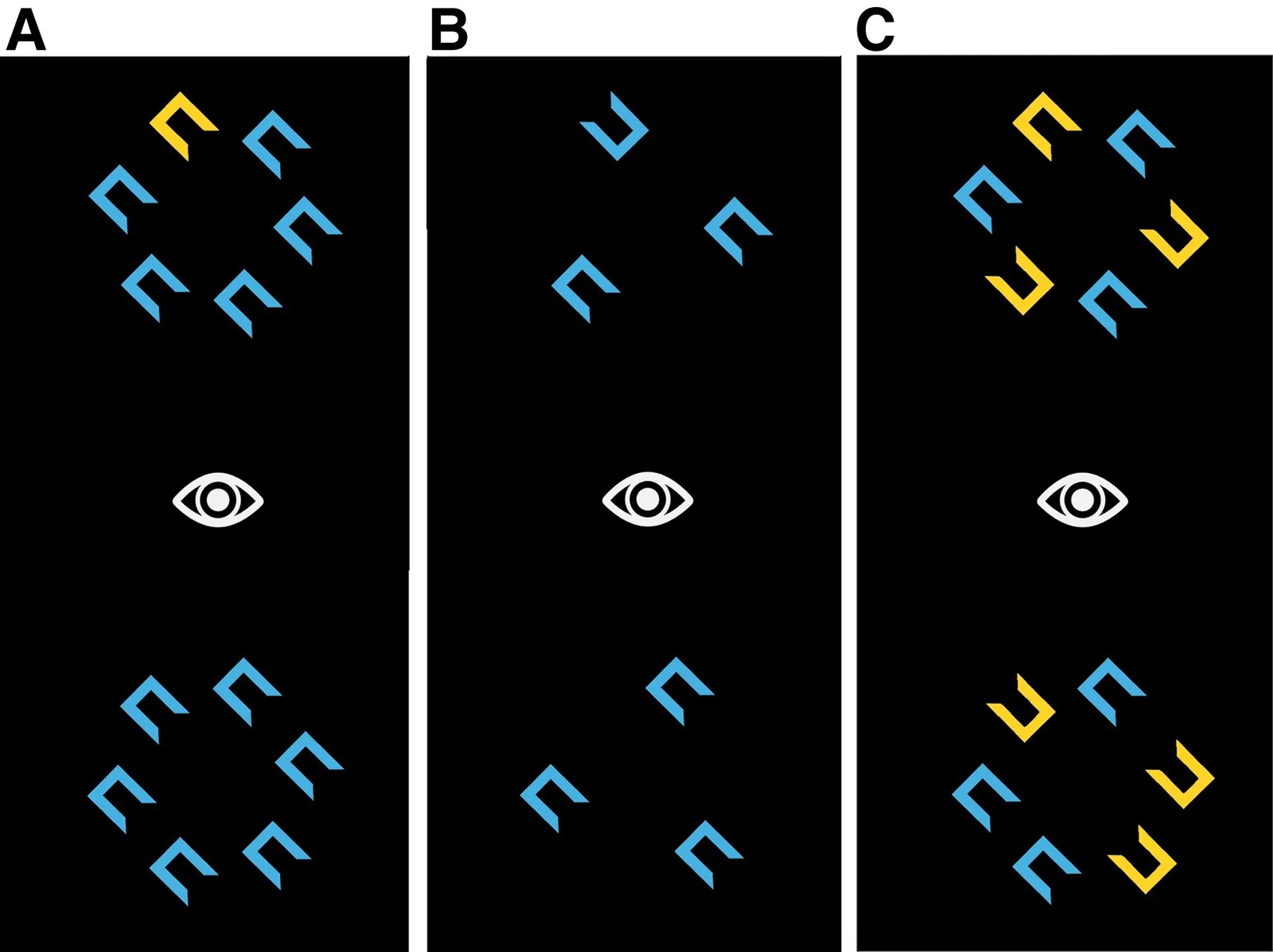
**Visual search tasks (A, Easy-Feature; B, Hard-Feature; and C, Conjunction) in the vertical axis condition**. Each panel represents the arrangement of the items in the 12-item Set Size (**A, C**) and 6-item Set Size (**B**). In the horizontal condition, the item wheels were positioned to the left and right of the eye image in a similar manner.

Trials began with the appearance of an eye picture at the centre of the screen, which participants had been instructed to fixate. After 1000 ms, the stimulus array appeared. Participants were instructed to press the left (right) arrow button of the keyboard with their left (right) index finger as fast as possible if they detected (or not) the target. In the 12-item Set Size, the stimulus array was arranged in two wheels of 6 items each (Ø 4°) flanked at ±6° from the centre of the screen. To avoid perfect symmetry, one of the two wheels was slightly rotated clockwise (or counterclockwise) in a pseudo-random manner. The most eccentric stimulus was located at ±7.88° from the centre of the screen and the closest one at ±4.12°. In each target-present condition, the 12 positions were tested once in addition to 3 top-wheel and 3 (non-symmetrical) bottom-wheel positions. In the six-item Set Size, a similar design was used, but the stimuli were arranged in two equilateral triangles to preserve an equal relative distance between each stimulus.

From this task, we analysed RT, *d*ʹ (sensitivity) and *c* criterion (bias) using a log-linear correction method for extreme proportions.^71^ Search Slopes (which reflect search efficiency) were computed by dividing the difference in RT in the 6 and 12 item conditions by the difference in Set Size (i.e. 6).

##### Multiple-target search: Bells test

This paper-based test requires the participant to circle with a pencil the 35 targets (black ink drawings of bells) embedded in 280 distractors (familiar figures such as houses, horses) on a landscape-oriented A4 paper sheet as fast as possible.^72^ The targets were pseudo-randomly arranged in seven columns containing five bells each. Search efficiency was modelled using a QoS score^70^ computed as follows:


$$\mathrm{QoS}=\;\frac{\mathrm{Correct}\;\mathrm{responses}}{\mathrm{Total}\;\mathrm{number}\;\mathrm{of}\;\mathrm{targets}}\times \;\frac{\mathrm{Correct}\;\mathrm{responses}}{\mathrm{Total}\;\mathrm{duration}}$$

Organization of search (reflecting executive functioning) was analysed using five parameters: (i) Total Distance (Euclidean length of the search path); (ii) Intersection Rate (ratio of total number of search path intersections to the amount of bell markings); (iii) IC distance (mean Euclidean distance between two consecutively cancelled targets); (iv) Best *R* (highest Pearson correlation between the position of marked bell locations, separately for *x* and *y* coordinates, relative to the order in which they were marked); and (v) Radial Strategy (Pearson correlation between rank number of each bell and the distance from that bell to the centre of the page).^73,74^ Positions of the stimuli were re-scaled within a circle (Ø 15) to ensure a similar distance between the centre and each border of the page for the Radial Strategy parameter.

#### Cognitive testing

Participants completed the MoCA, Parkinson’s Disease Questionnaire 39 (PDQ-39) and National Eye Institute 25-item Visual Function Questionnaire (NEI-VFQ 25). The third paper-based version of the MoCA was used to minimize potential test–retest effects from prior exposure to versions 1 or 2 (e.g. during clinical settings and/or other research projects).^75^ From this assessment, we obtained both the overall (global) score and individual subdomain scores. Fatigue was measured using the Visual Analogue Fatigue Scale.^76^ This instrument uses a 10-point Visual Analogue Scale where lower numbers indicate greater levels of fatigue.

### Data analysis

Analyses and illustrations were computed using SPSS, MATLAB, JASP and Jupyter Notebooks. Regarding oculomotor and visual search tasks, analyses of covariance (ANCOVAs) were used with a similar design: *Group* (PwP, PwPSP and AMC) as between-subject factor; Conditions (Saccade task: *Direction*; SPEM task: *Axis*; Visual search task: *Axis*, *Set Size*, *Target Presence* and *Task*) as within-subject factors; and *Age* as a covariate, with a separate dependent variable for each analysis (Saccades: *Amplitude gain*; SPEM: *Velocity gain*; Visual search: *dʹ*, *c criterion*, *RT*, *Intercept* and Slope). Estimated marginal means and standard errors were reported. Of note, the ‘Target Presence’ condition did not enter the ANCOVA for *dʹ* and *c criterion* parameters nor ‘Set Size’ for *Slope* and *Intercept* ANCOVAs due to the specific computational methods used for these parameters. A Greenhouse–Geisser correction was applied in case of sphericity violation. For the sake of clarity, we reported main effects and only significant interactions when the *Group* factor was highlighted. In the *post hoc* analyses, if no information is provided for a particular comparison (e.g. between the Parkinson’s disease and AMC groups), it indicates that no significant difference was found. Alpha was 0.05 and Holm–Bonferroni corrections were applied to *P*-values of *post hoc t*-tests to control for multiple comparisons.

Analyses of the MoCA and the Bells test were conducted in three steps: (i) dependent variables were compared between the groups using ANOVA and Holm–Bonferroni corrected independent samples *t*-tests; (ii) receiver operating characteristic (ROC) curves were computed for variables showing homoscedasticity of variance between the PwPSP and PwP groups; and (iii) ROC discriminability was interpreted using *AUC*, *Sensitivity* (*Se*) and *Specificity* (*Sp*). According to clinical standards,^77^  *AUC* values were classified as follows: 0.5–0.6 (failed), 0.6–0.7 (worthless), 0.7–0.8 (poor), 0.8–0.9 (good) and >0.9 (excellent). A parameter was considered clinically useful if the sum of *Se* and *Sp* exceeded 1.5.^78^

## Results

### Oculomotor behaviour

Oculomotor data were collected from 26 AMC, 18 PwPSP and 21 PwP due to technical issues with eyeglasses and/or signal noise. ANCOVA performed on saccadic gain revealed a main effect of *Group* (*F*(2,59) = 14.34, *P* < 0.001, $\eta_{p}^{2}$ = 0.33) and a Group*Direction interaction (*F*(6,177) = 9.87, *P* < 0.001, $\eta_{p}^{2}$ = 0.25). *Post hoc t*-tests indicated that the PSP group was significantly affected along the vertical plane (Upward: 0.60 ± 0.05; Downward: 0.52 ± 0.04) when compared with the Parkinson’s disease (Upward: 0.87 ± 0.05; Downward: 0.84 ± 0.04) and AMC (Upward: 0.88 ± 0.04; Downward: 0.91 ± 0.04) groups (*P* < 0.01). Visualizations of these data can be found in Supplementary Fig. 1, along with an analysis of smooth pursuit.

### Single-target visual search

We excluded one PwPSP who was unable to complete all the tasks. Two PwPSP and one PwP patient with very low accuracy (50, 51.39 and 16.67%, respectively) were also excluded. Analyses were thus performed on data for 25 PwPSP, 27 PwP and 27 AMC.

#### Sensitivity and bias

ANCOVA conducted on *dʹ* revealed a main effect of Group (*F*(2,75) = 10.94, *P* < 0.001, $\eta_{p}^{2}$ = 0.23), without any interaction. *Post hoc t*-tests indicated that PwPSP scored significantly lower (3.00 ± 0.08) than PwP (3.33 ± 0.08; *P* < 0.02) and AMC (3.54 ± 0.08; *P* < 0.001). A positive *c criterion* in each group (AMC: 0.09 ± 0.02, PwP: 0.09 ± 0.02, PwPSP: 0.12 ± 0.03) indicated that participants adopted a conservative strategy to solve the task (more likely to say the target is absent). There were no main effects, but a significant Set Size*Axis*Group interaction emerged (*F*(2,75) = 5.81, *P* = 0.005, $\eta_{p}^{2}$ = 0.13). *Post hoc t-*tests revealed an opposite pattern between PwPSP and AMC across the vertical and horizontal axes. For the vertical axis, *c criterion* in PwPSP significantly increased with the number of distractors (6 items: 0.06 ± 0.03; 12 items: 0.15 ± 0.04; *P* < 0.05), whereas this was not the case in the AMC group (6 items: 0.08 ± 0.03; 12 items: 0.09 ± 0.03; *ns*). In contrast, AMC participants showed a *c criterion* close to zero at the 6-item Set Size along the horizontal axis (0.03 ± 0.02) that significantly increased at 12 items (0.14 ± 0.03, *P* = 0.002) while PwPSP remained at a high value whatever the Set Size (6 items: 0.15 ± 0.02; 12 items: 0.12 ± 0.04; *ns*). PwP did not show any *Set Size* effect for either axis.

#### Reaction times

ANCOVA on RTs revealed main effects of *Age* (*F*(1,75) = 5.43, *P* = 0.02, $\eta_{p}^{2}$ = 0.07) and *Group* (*F*(2,75) = 32.08, *P* < 0.001, $\eta_{p}^{2}$ = 0.46). As depicted in Fig. 2, *post hoc t*-tests indicated that PwPSP was strikingly decoupled from both other groups (AMC: 938.10 ± 115.48; PwP: 1209.26 ± 117.03; PwPSP: 2230.92 ± 121.86, *P* < 0.001) regardless of task/condition. There was also a significant Task*Set Size*Target Presence*Group interaction (*F*(4,150) = 3.34, *P* = 0.01, $\eta_{p}^{2}$ = 0.08). In the Conjunction task, *post hoc t*-tests indicated that the three groups showed a significant increase in RTs when additional distractors were displayed both in the target present (AMC: +293.58 ± 57.61 ms, PwP: +295.24 ± 58.39, PwPSP: +619.43 ± 60.80; *P* < 0.001) as well as the target absent (AMC: +554.95 ± 64.45, PwP: +646.28 ± 65.32, PwPSP: +1208.61 ± 68.01; *P* < 0.001) conditions. In the Hard-Feature task, the same pattern emerged but only in PwP (target present: +167.67 ± 40.03, *P* < 0.001; target absent: +210.04 ± 65.52; *P* = 0.002) and PwPSP (target present: +258.61 ± 41.68, target absent: +473.70 ± 68.23; *P* < 0.001) groups. Regarding the Easy-Feature task, only PwPSP showed an increase of RTs in the target-present condition (+32.66 ± 12.09; *P* = 0.009). Finally, a significant Target Presence*Axis*Group interaction was observed (*F*(2,75) = 8.72, *P* < 0.001, $\eta_{p}^{2}$ = 0.19). While all groups showed an increase in RTs in the target-absent condition whatever the axis, *post hoc t*-tests revealed that this rise reached significance only in the two groups of patients when they faced the vertical condition (PwPSP: +307.41 ± 51.11; PwP: +107.64 ± 49.09; *P* < 0.05).

**Figure 2:**
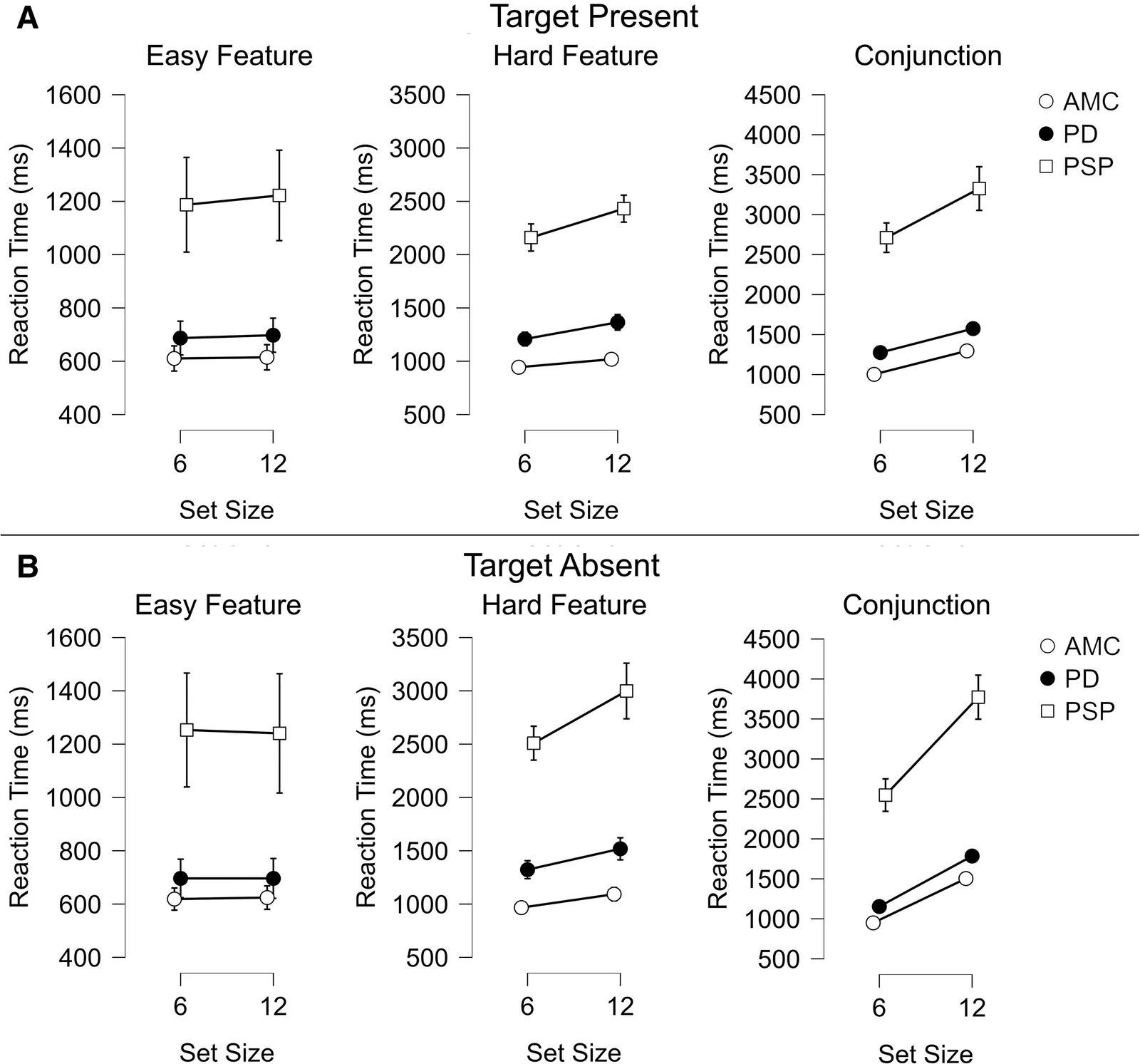
**Mean RTs in single-target search tasks in each group.** Data were analysed with a 3 × 2 × 2 × 2 mixed ANOVA, with a between-participant factor of Group (AMC: *N* = 27, Parkinson’s disease: *N* = 28, PSP: *N* = 28); within-participant factors of Axis (horizontal and vertical), Set Size (6 and 12 items), Target Presence (present and absent) and Task (Easy-Feature, Hard-Feature and Conjunction); and a covariate of Age. The figure illustrates the significant Task*Set Size*Target Presence*Group interaction (*F*(4,150) = 3.34, *P* = 0.01, $\eta_{p}^{2}$ = 0.08). (**A**) Target-present trials and (**B**) Target-absent trials. Line style represents the Set Size conditions (straight: 6 items; dashed: 12 items). In the Easy-Feature task, the effect of Set Size was significant in the Parkinson’s disease group in the Target-Present condition (**A**, left panel; *F* = 5.15, *P* = 0.032), but not the Target-Absent condition (**B**, left panel; *F* = 0.1, *P* = 0.98). There was no effect of Set Size in the other groups (AMC: *F* = 1.55, *P* = 0.22; PSP: *F* = 0.45, *P* = 0.51). In the Hard-Feature task, all three groups showed a significant effect of Set Size in the Target Present (**A**, centre panel; AMC: *F* = 26.64, *P* < 0.001; Parkinson’s disease: *F* = 38.09, *P* < 0.001; PSP: *F* = 16.44, *P* < 0.001) and Target Absent (**B**, centre panel; AMC: *F* = 28.139, *P* < 0.001; Parkinson’s disease: *F* = 30.02, *P* < 0.001; PSP: *F* = 18.97, *P* < 0.001) conditions. In the Conjunction task, analysis of simple main effects indicated that all three groups showed a significant effect of Set Size in the Target Present (**A**, right panel; AMC: *F* = 217, *P* < 0.001; Parkinson’s disease: *F* = 112, *P* < 0.001; PSP: *F* = 38.5, *P* < 0.001) and Target Absent (**B**, right panel; AMC: *F* = 217.9, *P* < 0.001; Parkinson’s disease: *F* = 112, *P* < 0.001; PSP: *F* = 38.5, *P* < 0.001) conditions. Error bars show 95% CIs.

#### Intercepts

ANCOVA conducted on Intercepts revealed main effects of *Age* (*F*(1,75) = 5.30, *P* = 0.02, $\eta_{p}^{2}$ = 0.07) and *Group* (*F*(2,75) = 24.39, *P* < 0.001, $\eta_{p}^{2}$ = 0.39). The PwPSP group showed a very large Intercept (1585.47 ± 98.24) when compared with PwP (876.59 ± 94.35; *P* < 0.001) and AMC (673.05 ± 93.10; *P* < 0.001). No interaction involving the *Group* factor emerged.

#### Slopes

Results of the Slopes in each group are depicted in Fig. 3. ANCOVA showed main effects of *Task* (*F*(2,135.87) = 3.84, *P* < 0.05, $\eta_{p}^{2}$ = 0.05), *Axis* (*F*(1,75) = 6.60, *P* = 0.01, $\eta_{p}^{2}$ = 0.08), *Group* (*F*(2,75) = 22.71, *P* < 0.001, $\eta_{p}^{2}$ = 0.38) and a Task*Target Presence*Group interaction (*F*(4,150) = 3.34, *P* = 0.01, $\eta_{p}^{2}$ = 0.08). *Post hoc t*-tests indicated that in the Easy-Feature task, Slopes were clustered around zero for all groups in the target present (PwPSP: 5.44 ± 2.02; PwP: 2.14 ± 1.94, AMC: 0.75 ± 1.91) and target-absent conditions (PwPSP: −1.87 ± 1.98; PwP: −0.23 ± 1.90, AMC: 0.92 ± 1.88) without any difference between the groups (*P* > 0.29). Inspection of Fig. 3A suggested that PwPSP had search Slopes that differed from 0 along the vertical axis. A one-sample *t*-test confirmed a significant effect of *Slope* (10.69 ± 5.38, *P* = 0.01). There was no effect on the horizontal axis (−5.11, SEM: 8.07, *ns*). In the Hard-Feature and the Conjunction tasks, the Slopes in the PwPSP group were dramatically steeper than the Slopes for the other groups. In the target-present condition, a significant difference emerged between PwPSP and AMC in both the Hard-Feature (+30.55 ± 9.58, *P* = 0.006) and Conjunction (+54.31, ±13.97, *P* < 0.001) tasks, while PwP differed only from PwPSP in the Conjunction task (+54.03, ±14.25, *P* < 0.001). In the target-absent condition, the PwPSP group had steeper Slopes than both other groups in the Hard-Feature and Conjunction tasks (all *P* < 0.05).

**Figure 3:**
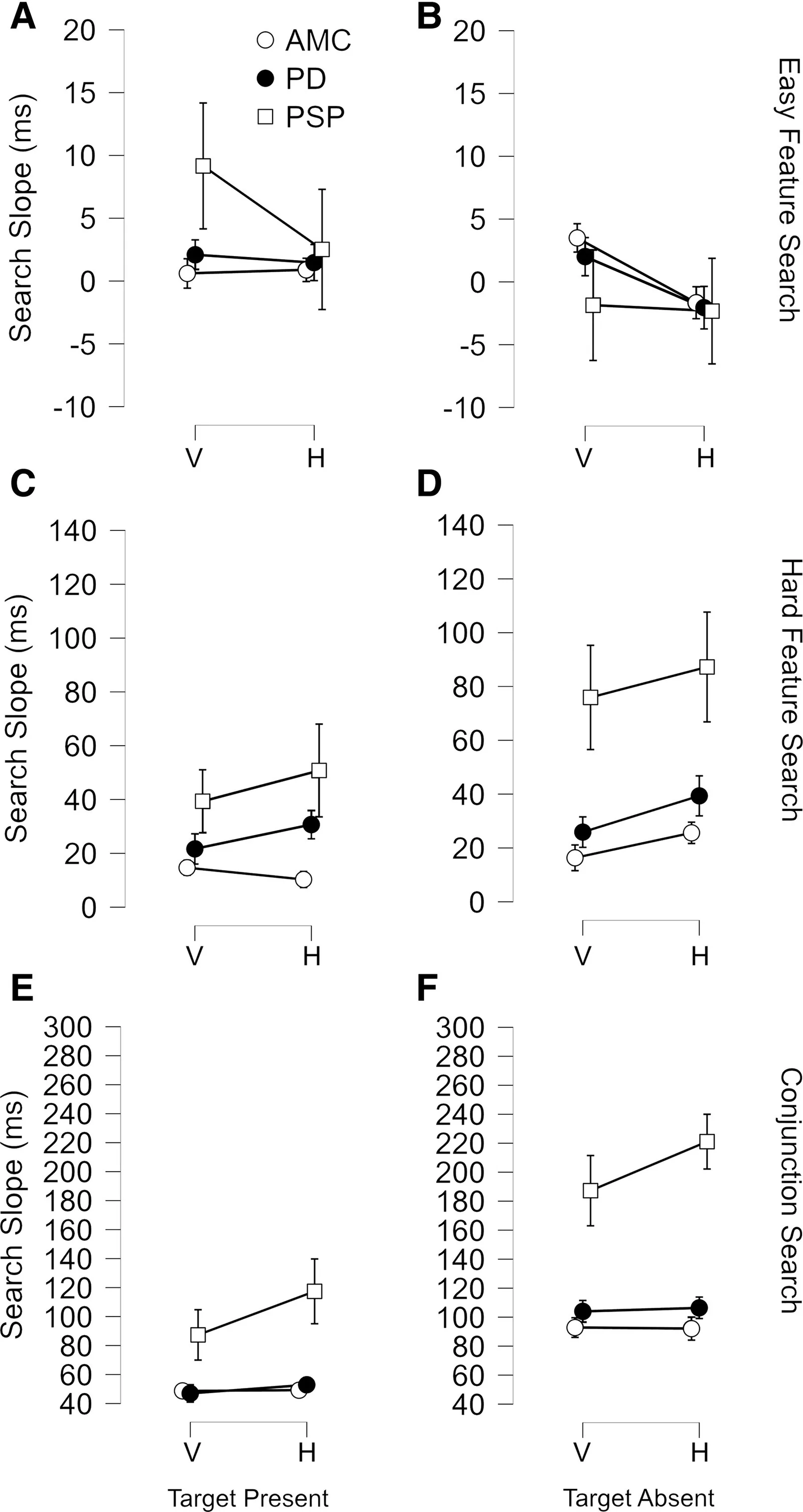
**Mean Slopes in single-target search tasks in each group. (A, B**) Slopes in the Easy-Feature task; (**C, D**) Hard-Feature task; (**E, F**) Conjunction task. Left panels: Target-Present condition; right panels: Target-Absent condition. Each graph illustrates separately the Slopes for vertical (V) and horizontal (H) conditions. AMC: *N* = 27; Parkinson’s disease: *N* = 28; and PSP: *N* = 28. ANCOVA revealed a Task*Target Presence*Group interaction (*F*(4,150) = 3.34, *P* = 0.01, $\eta_{p}^{2}$ = 0.08). Simple main effects in the Target-Present condition showed main effects of Group on the Hard-Feature(*F* = 5.1, *P* = 0.008) and Conjunction tasks (*F* = 9.64, *P* < 0.001), but not the Easy-Feature task (*F* = 1.48, *P* = 0.008). Similarly, in the Target-Absent condition, there were main effects of Group in the Hard-Feature (*F* = 7.2, *P* < 0.001) and Conjunction tasks (*F* = 27.6, *P* < 0.001), but not the Easy-Feature task (*F* = 0.52, *P* = 0.008). Error bars show 95% CIs.

### Multiple-target visual search and MoCA

Data on the Bells test were collected for all participants, but one PwP and five PwPSP were unable to complete the MoCA (mainly due to their inability to use a pen for tasks involving fine motor skills and/or severe language production impairment).

#### Comparisons between the groups

One PwP and five PwPSP were unable to complete the MoCA. ANOVA and means are summarized in Table 2. PwPSP had drastically reduced performance in the Bells test. All *t*-tests comparing PwPSP to both other groups were significant for each parameter (*P* ≤ 0.02; Cohen’s *d* range: 0.54–2.17), consistent with profoundly disordered visual search in PSP. PwP had shorter IC distance and lower Total Distance scores than AMC, but neither effect was statistically significant following Holm–Bonferroni correction (ICD: PwP: 5.07 ± 0.28; AMC: 5.74 ± 0.27; *t* = 1.66; *P* = 0.1, Cohen’s *d*: 0.45; Total Distance PwP: 161.29 ± 9.44; AMC: 183.22 ± 9.57; *t* = 1.69; *P* = 0.095, Cohen’s *d*: 0.44).

**Table 2:** Performance in the multiple-target search task (Bells test)

	Group			Levene’s test PSP versus PD	*F*-value	*η* ^2^	*Post hoc t*-test
	AMC (*N* = 27)	PwP (*N* = 28)	PwPSP (*N* = 28)				
Multiple target search (Bells test)							
Omission (%)	5.19 ± 1.18	6.12 ± 1.80	13.57 ± 3.13	*P* = 0.01	4.34	0.098	ab*
Time (s)	110.81 ± 5.74	128.64 ± 7.99	238.11 ± 20.58	*P* < 0.05	26.94	0.402	ab***
QoS	0.31 ± 0.02	0.28 ± 0.03	0.13 ± 0.01	ns	22.7	0.362	ab***
Best R	0.87 ± 0.03	0.81 ± 0.04	0.47 ± 0.05	ns	25.54	0.390	ab***
Radial strategy	0.08 ± 0.02	0.11 ± 0.02	0.20 ± 0.03	ns	6.57	0.141	a*b**
Total distance	183.22 ± 9.57	161.29 ± 9.44	210.84 ± 8.44	ns	7.44	0.157	a***
IC distance	5.74 ± 0.27	5.07 ± 0.28	7.37 ± 0.30	ns	17.58	0.305	ab***
Intersection rate	1.56 ± 0.51	1.17 ± 0.44	4.97 ± 0.64	*P* < 0.01	15.23	0.276	ab***

Data are presented as mean ± SD. The experimental unit for all analyses was the individual participant. *Post hoc t*-tests were Holm–Bonferroni corrected for familywise error. Significant differences are highlighted by the letters ‘a’ (PwPSP versus PwP), ‘b’ (PwPSP versus AMC) and ‘c’ (PwP versus AMC). *Corrected *P* < 0.05; **corrected *P* < 0.01; **corrected *P* < 0.001.

In the MoCA, both patient groups (PwPSP: 21.26 ± 0.68 and PwP: 25.48 ± 0.55) differed significantly from AMC participants (25.52 ± 0.53). PwPSP also had significantly lower scores than the PwP group (*t* = 4.85; *P* < 0.001; Cohen’s *d* = 1.38).

#### Receiver operating characteristic analyses

We excluded the Omission, Time and Intersection Rate parameters due to the inequality of variances. ROC analyses were performed on the remaining variables, and all sub-dimensions in the MoCA test. Table 3 illustrates the results, with bold values indicating parameters in which homoscedasticity was verified.

**Table 3:** ROC analyses comparing PSP (*N* = 28) and Parkinson’s disease (*N* = 28) groups on the multiple-target search and MoCA

	AUC	*Z* value	Specificity	Sensitivity	Optimal cut-off
Multiple target search (Bells test)					
QoS	**0.90**	8.93***	**0.96**	**0.82**	**0.16**
Best *R*	**0.82**	5.53***	**0.75**	**0.86**	**0.78**
Radial strategy	**0.70**	2.81**	**0.54**	**0.86**	**0.08**
Total distance	**0.80**	4.45***	**0.79**	**0.89**	**168.12**
IC distance	**0.88**	7.06***	**0.75**	**1.00**	**5.09**
MoCA					
Global score	**0.84**	5.99***	**0.79**	**0.82**	**24**
Delayed recall	**0.70**	2.77**	**0.43**	**0.96**	**4**
Language	0.70	2.89***	0.96	0.36	2
Visuospatial/executive	0.75	3.43***	0.89	0.55	3
Attention	0.65	1.97*	0.96	0.36	5
Abstraction	0.55	1.45, ns	N/A	N/A	N/A
Orientation	0.56	0.94, ns	N/A	N/A	N/A
Naming		*ROC: non-applicable*			

Bold values indicate parameters for which homoscedasticity was verified. The experimental unit was the individual participant. ns, non-significant. * = *P* < 0.05, ** = *P* < 0.01, *** = *P* < .001.

As shown in Fig. 4A, PwPSP had a marked deficit in Bells test performance compared with PwP or AMC. ROC analysis indicated that QoS demonstrated excellent ability to discriminate PSP from Parkinson’s disease [AUC = 0.90, 95% DeLong confidence interval (CI) 0.82–0.99; sensitivity = 82%, specificity = 96% at the optimal cut-off of 0.164; Fig. 4B and C]. IC distance also demonstrated excellent discrimination between PSP and Parkinson’s disease (AUC = 0.88, 95% DeLong CI 0.74–0.1; sensitivity = 100%, specificity = 75% at the optimal cut-off of 5.09; Fig. 4B and C). The (*Sp* + *Se*) of QoS and IC distance exceeded the threshold of 1.5 for clinical utility (1.78 and 1.75, respectively) (Table 3, Fig. 4B and C).

**Figure 4:**
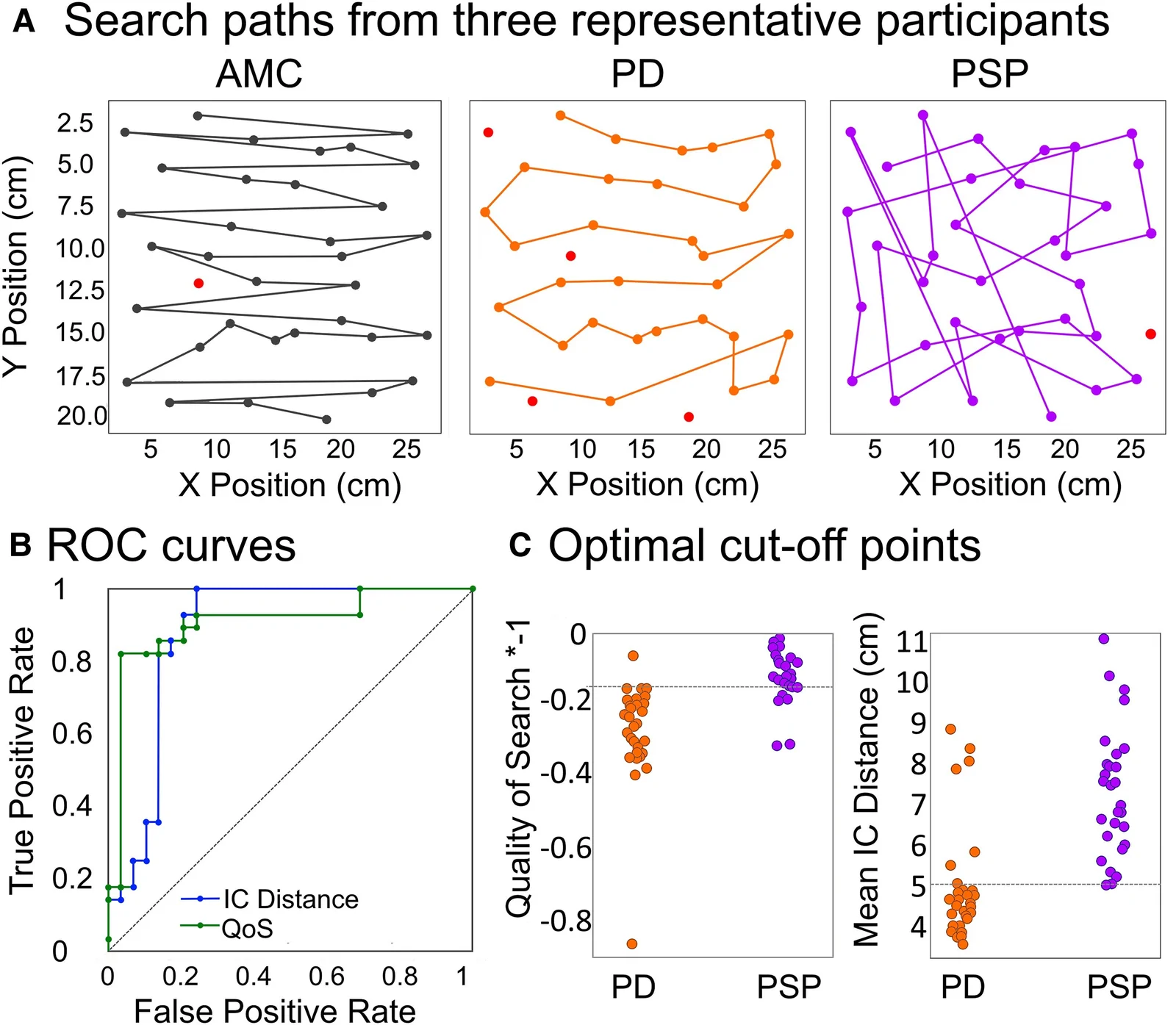
**Results in the multiple-target search task (Bells test).** (**A**) Search path in three representative participants. AMC: *N* = 27; PD = Parkinson’s disease: *N* = 28; PSP, *N* = 28. The red spot indicates a missed target. (**B**) ROC illustration of the two best discriminative parameters between PSP and Parkinson’s disease and the corresponding AUC. The experimental unit for analyses was the individual participant. QoS demonstrated excellent ability to discriminate PSP from Parkinson’s disease (AUC = 0.90, 95% DeLong CI 0.82–0.99; *Se* = 82%, *Sp* = 96% at the optimal cut-off of 0.164). IC distance also demonstrated excellent discrimination between PSP and Parkinson’s disease (AUC = 0.88, 95% DeLong CI 0.74–0.1; *Se* = 100%, *Sp* = 75% at the optimal cut-off of 5.09). (**C**) Performance of the PwPSP (*N* = 28) and PwP (*N* = 28) on these two parameters; the dashed line represents the optimal cut-off. Filled spots indicate individual patient scores. Note that the QoS value has been multiplied by −1 to directly compare both parameters (highest scores illustrate a deficit).

The MoCA global score also discriminated between PwP and PwPSP, but with a lower *AUC* value (0.82) and a lower combined *Sp* and *Se* measure (1.61) compared with QoS or IC distance in the Bells test. Furthermore, only four MoCA subdomains succeeded in distinguishing PwPSP from PwP. Of these, three subdomains (Visuospatial-Executive, Language and Attention) demonstrated very low *Se* (range: 0.36–0.55) and lacked homoscedasticity. The Delayed Recall subdomain met the homoscedasticity criterion but exhibited low *Sp* (0.43). In contrast, most parameters of the Bells test (except for Radial Strategy) displayed a more favourable balance between *Sp* (range: 0.75–0.96) and *Se* (range: 0.82–1.00).

## Discussion

This study investigated visuospatial attention in PwPSP and PwP. As predicted by our primary hypothesis, PwPSP were significantly slower and less efficient than PwP and AMC participants for single-target search. This impairment was more severe for Conjunction search than Hard-Feature search, and Easy-Feature search exhibited a horizontal–vertical anisotropy, such that search was significantly less efficient along the vertical axis compared with the horizontal axis. Multiple-target search (the Bells test) was also affected by PSP, as PwPSP exhibited a profound impairment of search organization compared with PwP and AMC. ROC analysis using search efficiency parameters derived from the Bells test classified PwPSP and PwP with 90% accuracy, outperforming the MoCA. Consistent with the typical presentation of PSP,^79,80^ PwPSP showed a deficit of saccadic eye movements and SPEMs along the vertical plane, while performance along the horizontal meridian fell in the normal range.

In the single-target search tasks, each of which engages partially distinct cognitive processes, ANCOVAs performed over each metric of interest revealed group differences that were task-independent (Intercepts, *dʹ* and *c criterion*) and task-specific (RTs and Slopes). The extremely slow RT in PwPSP is likely to be due to a combination of impaired attentional processes and akinesia that slows initiation of the motor response. Indeed, task-independent effects also highlighted a general increase in the Intercept in PwPSP regardless of condition, consistent with a general poorer movement quality. In contrast, exaggerated search Slopes in PwPSP in Hard-Feature and Conjunction search tasks indicate that the efficiency of attentional processing was compromised in this group. The finding that the magnitude of the impairment of visual search was similar for horizontally and vertically presented arrays but was larger for more complex search tasks is also consistent with the conclusion that the deficits are due to problems with attentional processes, rather than due to an inability to move the eyes to targets *per se*. At a systems level, reduced visual search efficiency may be associated with degeneration of the superior colliculus and adjacent tectal nuclei characteristic of PSP. These structures are critical for integrating visual and motor signals across cortical and subcortical pathways and form a key node in salience maps that guide spatial attention and eye movements. Their dysfunction disrupts the integration necessary for prioritizing competing visual inputs, suggesting that a key source of the visual search deficits observed in PwPSP is impaired computation *of relative saliency* between stimuli. Impaired salience computations will slow down all searches but should have a larger effect when the target is low salience (as in the Conjunction search) than when it is higher salience (i.e. the Hard-Feature search task), and lead to less organized search, which is the pattern observed in PwPSP. Search efficiency was also reduced in the Easy-Feature task, but only along the vertical axis. More specifically, a search Slope of 0 along the horizontal axis indicates that the high salience targets ‘popped-out’ of the display, consistent with an efficient, parallel search. In contrast, PwPSP had Slopes of ∼11 ms/item along the vertical, indicating a switch to a less efficient, serial search strategy. This finding is reminiscent of previous observations of impaired exogenous, covert orienting along the vertical axis in PwPSP,^30,31^ and evidence that experimental constraints of oculomotility can produce deficits in Feature search tasks.^81,82^ This cannot be explained in terms of the physical limitation on vertical eye movements, because Easy-Feature search is solved without using eye movements. Rather, it suggests that exogenous attentional processes are particularly sensitive to disruptions of the oculomotor system, consistent with the idea of a tight coupling between exogenous attention and oculomotor control.^83^

PwPSP were significantly less sensitive and more conservative than the other groups. However, given that a *d′* value around 3 or higher indicates excellent discrimination with minimal signal–noise overlap, and *c criterion* below 0.2 (in absolute value) reflects only a slight liberal or conservative tendency, these data suggest relatively subtle differences in perceptual performance between the PwPSP and other groups. Importantly, the preserved perceptual performance in PwP rules out the possibility that the impaired search performance is caused by problems with visual functioning.

There were no statistically significant differences in RT between the AMC and PwP in the Easy-Feature and Conjunction search tasks. However, on the Hard-Feature search task, PwP showed significant set-size effects, whereas the AMC group did not. On first inspection, this result may appear inconsistent with previous evidence for a feature search deficit in PwP.^65,84-86^ However, not all studies observe disrupted feature search in PwP,^33,68,87,88^ and an anti-dopaminergic drug does not affect feature search in healthy adults.^89^ Horowitz *et al.*^90^ argued that the probability of observing a visual search deficit in PwP depended on the saliency of the target. They proposed that dopaminergic depletion could induce a weaker representation of salient signals, thus prolonging selection time, but only when targets are relatively low salience. This deficit could be alleviated with top–down knowledge. In the current study, patients always knew target identity but targets in the Hard-Feature task were mirror-reversed, which is a relatively low salience type of feature singleton that typically increases target identification process time in healthy subjects.^91^ Furthermore, colour vision and orientation tuning can be affected in Parkinson’s disease,^92^ but the latter could have a stronger impact on search.^85^ The subtle visual search deficit observed in the PwP in the Hard-Feature search task may therefore reflect a subtle deficit of saliency computation that is only observed when target salience is relatively low.

Performance in the Bells test was impaired in PwPSP compared with PwP and AMC participants. PwPSP omitted a significantly higher proportion of bells (∼14%) and required twice as much time (around 4 min) to complete the task, resulting in a significantly lower QoS score. These findings align with functional magnetic resonance imaging studies, demonstrating that cancellation task performance is closely linked to brain activity in networks supporting visuospatial attention, motor planning and execution, eye movements and executive control,^93-96^ which are functions known to be impaired in PwPSP. Accordingly, the QoS metric appears to be sensitive to dysfunction in three of the four diagnostic domains highlighted by the MDS-PSP criteria: oculomotility, movement quality and visuospatial cognition. ROC analyses were consistent with this conclusion: QoS was highly effective in distinguishing PSP from Parkinson’s disease (*AUC*: 0.90, *Se*: 0.82, *Sp*: 0.96), outperforming the MoCA global score (*AUC*: 0.84, *Se*: 0.82, *Sp*: 0.79). Additionally, PwPSP showed the lowest Best *R* value, and highest IC distance and Intersection Rate, indicative of a highly disorganized search, consistent with the previous finding that PwPSP patients are significantly less likely to adopt an effective search strategy than PwP.^56^ PwPSP patients had a higher Radial Strategy score when compared with both other groups, suggesting a tendency to scan for targets around the edges of the array before moving inwards. However, Radial Strategy (0.20) was still only half of the Best *R* (0.47), suggesting a general disorganization of search rather than the adoption of a specific strategy. ROC analyses indicated that IC distance was the second-best parameter at classification of PwP and PwPSP (*AUC*: 0.88) but was extremely sensitive to PSP (*Se*: 1, *Sp*: 0.75). The high sensitivity and specificity of the QoS and IC distance indicate the potential utility of the Bells test for differentiating PSP and Parkinson’s disease.

Overall, these data demonstrate that the Bells test can differentiate PSP and Parkinson’s disease with a high degree of sensitivity and specificity, and thus shows excellent potential to facilitate differential diagnosis. The Bells test also offers several other advantages. For example, while some of the parameters depend on time duration (Completion Time and QoS), this is not the case for IC distance. This distinction is important because it allows us to reasonably assume that these latter measures reflect the pure effect of executive dysfunction, without being confounded by delays in motor response because successful performance on the Bells test also requires the integrity of both the oculomotor system and attentional/executive function. Although tau propagation differentially affects PSP subtypes, midbrain and frontal cortex dysfunction occur early in the pathogenesis, so the Bells test may have potential for facilitating earlier diagnosis. Furthermore, this test is free, short (mean completion time was ∼4 min for PwPSP) and easy to administer, and the QoS metric is relatively straightforward to compute, which is beneficial in clinical settings where time and/or resources may be limited. A quick guide to computing such analyses is included on OSF (see ‘Data Availability’ section). Visual search tasks are also highly flexible for test–retest sessions and can be adapted to assess additional dimensions such as set shifting (foraging tasks) or spatial memory (making the target marking invisible). Finally, as a visuospatial test, it does not require translation to other languages, so is easily adaptable to non-Anglophone cultures.

### Limitations

Most patients were on dopaminergic medication at the time of testing (26 PwP and 25 PwPSP). Given the limited effect of dopaminergic drugs on PSP, one might argue that the group-level differences we observed in cognitive performance are an indirect measure of responsiveness to dopamine, rather than reflecting a specific cognitive deficit in PSP. However, this explanation is unlikely for two reasons. First, the critical deficit was on a measure of visuospatial attention, which is relatively insensitive to dopamine agonists.^89^ Second, studies comparing the effect of dopaminergic medications on visual search and cancellation tasks in medicated and unmedicated parkinsonian patients have small and unreliable effects,^67,97^ or affect only the motor, and not cognitive, aspects of performance.^98^ A second limitation is that the PSP and Parkinson’s disease groups differed significantly in terms of age (*P* < 0.05) and time to diagnosis/onset of symptoms (*P* < 0.001), such that the PSP group were older but had received their diagnosis more recently. Ageing can modulate visual search performance in healthy populations,^99-102^ but its effect on response time is of the order of 2.8 ms/year,^102^ which cannot explain the profound delay observed in the study, and our conservative methodology, which included age as a covariate, controlled for this potentially confounding effect. It also should be kept in mind that PSP is characterized by a more rapid progression when compared with Parkinson’s disease, although the PDQ-39 indicated an overall similarity of health status in the two patient groups. Finally, patients were diagnosed clinically, and in some cases, this relied on expert clinical judgement rather than standardized formal diagnostic criteria. It is also important to note that while this study provides evidence for the validity of search tests for differentiating PSP and Parkinson’s disease, prospective trials in cohorts where there is diagnostic uncertainty are required to confirm the clinical usefulness of visual search tests.

## Conclusion

Tests that differentiate PSP and Parkinson’s disease early in disease progression are a high priority for patients, families and carers. This study reveals a profound impairment of visual search that appears to be highly specific to PSP; RTs and task completion time were markedly prolonged in PSP relative to the Parkinson’s disease and AMC groups, reflecting severe deficits in search efficiency in single- and multiple-target paradigms. Furthermore, PSP patients demonstrated highly disorganized search patterns, indicative of compromised attentional and executive processes. Critically, ROC analyses showed that the QoS metric derived from the Bells test excelled at differentiating PwPSP from PwP (*Se* 82%; *Sp* 96%; *AUC* 0.90), demonstrating the potential clinical utility of visual search assessments for early and reliable differentiation of these diseases.

## Supplementary Material

fcag422_Supplementary_Data

## Data Availability

The data that support the findings of this study are available on OSF at: https://osf.io/bzgxw/.
