# Supplementary material for: Visual search as a cognitive marker to discriminate progressive supranuclear palsy from Parkinson’s disease
Source: Brain Commun. 2026 Nov 3;8(6):fcag422. doi: 10.1093/braincomms/fcag422 (PMC13759987; doi:10.1093/braincomms/fcag422)
Supplement: Supplementary file 1 [file fcag422_supplementary_data.docx]

**Supplementary Material**

*Analysis of Smooth Pursuit*: The initial and final half-cycle were removed. The remaining 10 cycles were smoothed by removing blinks and saccades and then applying a Savitzky-Golay filter. We then identified the positive and negative peaks, calculated the unsigned difference between the two for each cycle, then averaged this difference across all 10 cycles. Pursuit Gain was calculated by dividing the magnitude of the mean unsigned difference by the actual target amplitude.

**Supplementary Figure 1**

Supplementary Figure 1: **Oculomotor data in the three groups (26 Age Matched Controls (AMC), 18 People with Progressive Supranuclear Palsy (PSP), and 21  people with Parkinson’s disease (PD).** ANCOVA performed on mean saccadic gain (actual saccade amplitude divided by the target displacement) revealed a main effect of *Group* (*F* _(2,59)_ = 14.34, *p* < .001, *η^2^_p_* = .33) and a *Group*Axis* interaction (*F* _(6,177)_ = 9.87, *p* < .001, *η^2^_p_* = .25). Post-hoc *t*-tests indicated that the PSP group had significantly reduced gain for downwards (Panel A; PSP: 0.52 ± .04; PD: 0.84 ± .04; AMC 0.91 ± .04) and upwards (Panel B; PSP: 0.60 ± .05; .PD: 87 ± 0.05; AMC.88 ± 0.04; *p* values <.01) saccades. However, there were so group differences for leftwards (Panel C) or rightwards (Panel D) movements.

Panel E shows Smooth Pursuit Eye Movements (SPEM) in a representative participant from each group in the low frequency, vertical condition. The grey dashed line represents the target's Y position over time, while the solid line indicates the eye's Y position over time. ANCOVA conducted on mean SPEM velocity gain revealed a main effect of *Group* (*F*_(2,59)_ = 3.72, *P* < .05, *η^2^_p_* = .11) and a *Group*Axis* interaction (*F*_(6,177)_ = 6.59, *P* < .01, *η^2^_p_* = .18). As illustrated in Panel E, PSP patients tended to maintain their gaze at the centre, failing to effectively track the target when it moved along the vertical axis. This resulted in a marked reduction in velocity gain along the vertical meridian (0.50 ± .04) as compared to both other groups (AMC: 0.69± .03; PD: 0.64± .03, *P* values ≤.01). In contrast, participants were almost indistinguishable along the horizontal plane ( p ≥. 75; *ns*) gains.
